# Supplementary figures and images for: Interface Effects on the Electronic and Optical Properties of Graphitic Carbon Nitride (g-C3N4)/SnS2: First-Principles Studies
Source: Materials (Basel). 2025 Feb 18;18(4):892. doi: 10.3390/ma18040892 (PMC11857837; doi:10.3390/ma18040892)

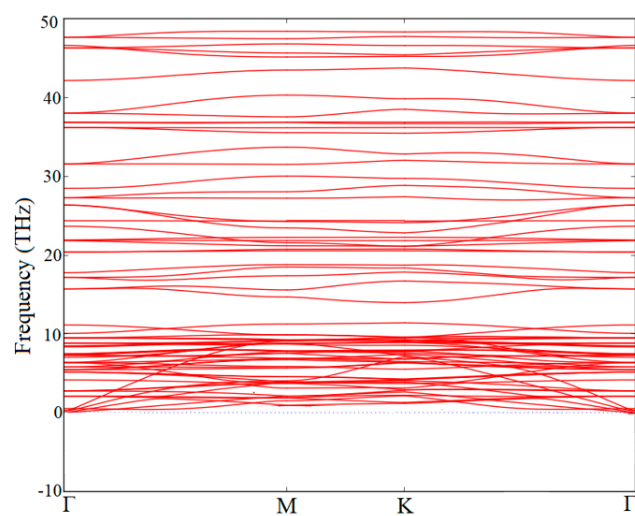

**Figure S1.** Phonon dispersion of g-C<sub>3</sub>N<sub>4</sub>/SnS<sub>2</sub>.

Supplement: Supplementary file 1 [file materials-18-00892-s001.zip › materials-3456613-supplementary.pdf]
